# AI-Powered Mental Health Virtual Assistants' Acceptance: An Empirical Study on Influencing Factors Among Generations X, Y, and Z

**DOI:** 10.7759/cureus.49486

**Published:** 2023-11-27

**Authors:** Turki Alanzi, Abdullah A Alsalem, Hessah Alzahrani, Norah Almudaymigh, Abdullah Alessa, Raghad Mulla, Lama AlQahtani, Reem Bajonaid, Abdulaziz Alharthi, Omar Alnahdi, Nouf Alanzi

**Affiliations:** 1 Department of Health Information Management and Technology, College of Public Health, Imam Abdulrahman Bin Faisal University, Dammam, SAU; 2 College of Medicine, King Saud University, Riyadh, SAU; 3 ‏College of Science and Humanities, Shaqra University, Shaqra, SAU; 4 College of Medicine, Al Faisal University, Riyadh, SAU; 5 College of Medicine, King Abdulaziz University, Jeddah, SAU; 6 College of Medicine, Imam Muhammad Ibn Saud Islamic University, Riyadh, SAU; 7 College of Pharmacy, Jazan University, Jazan, SAU; 8 Department of Public Health, Dr. Sulaiman AlHabib Hospital, Alkhobar, SAU; 9 Department of Clinical Laboratories Sciences, College of Applied Medical Sciences, Jouf University, Sakakah, SAU

**Keywords:** gen z, gen y, gen x, mental health, virtual assistants, chatbots, artificial intelligence

## Abstract

Study purpose: This study aims to analyze various influencing factors among generations X (Gen X), Y (Gen Y), and Z (Gen Z) of artificial intelligence (AI)-powered mental health virtual assistants.

Methods: A cross-sectional survey design was adopted in this study. The study sample consisted of outpatients diagnosed with various mental health illnesses, such as anxiety, depression, schizophrenia, and behavioral disorders. A survey questionnaire was designed based on the factors (performance expectancy, effort expectancy, social influence, facilitating conditions, and behavioural intention) identified from the unified theory of acceptance and use of the technology model. Ethical approval was received from the Ethics Committee at Imam Abdulrahman Bin Faisal University, Saudi Arabia.

Results: A total of 506 patients participated in the study, with over 80% having moderate to high experience in using mental health AI assistants. The ANOVA results for performance expectancy (PE), effort expectancy (EE), social influence (SI), facilitating conditions (FC), and behavioral intentions (BI) indicate that there are statistically significant differences (p < 0.05) between the Gen X, Gen Y, and Gen Z participants.

Conclusion: The findings underscore the significance of considering generational differences in attitudes and perceptions, with Gen Y and Gen Z demonstrating more positive attitudes and stronger intentions to use AI mental health virtual assistants, while Gen X appears to be more cautious.

## Introduction

Gen X, born between the early 1960s and early 1980s, experienced a significant portion of their lives without the ubiquity of digital technology. As they witnessed the transition from analog to digital, their perceptions of artificial intelligence (AI)-powered mental health interventions might differ from those of younger generations. Millennials, born between the early 1980s and mid-1990s (Gen Y), were among the first to embrace the digital era, witnessing the rapid evolution of online communication and digital platforms. Gen Z, born from the mid-1990s to the early 2010s, has grown up in a world immersed in digital technology and social media. This study recognizes that each generation possesses distinct characteristics shaped by their unique life experiences, technological exposure, and attitudes toward seeking mental health support [1,2].

The utilization of AI-powered virtual assistants in mental healthcare holds promise due to their potential to provide immediate and personalized support. These virtual assistants can engage users in conversational interactions, offering coping strategies, psychoeducation, and even crisis intervention [3]. Moreover, they operate with a level of anonymity that can reduce the stigma often associated with seeking traditional mental health services [4]. However, the acceptance of AI-powered mental health virtual assistants can be influenced by various factors, including familiarity with technology, attitudes toward mental health, concerns about privacy and data security, and preferences for human interaction in sensitive contexts [5-7].

This study recognizes the need to assess the generational nuances that might impact the adoption of AI-powered mental health virtual assistants. Gen X, for instance, might exhibit a mix of curiosity and skepticism about the efficacy of AI in addressing mental health concerns. Their preferences for traditional forms of therapy might clash with the automated nature of chatbot interventions. On the other hand, millennials, who have navigated the transition from traditional forms of communication to digital platforms, might be more inclined to explore AI-powered solutions. Their familiarity with technology could make them early adopters of virtual assistants, provided they perceive the interventions as credible and effective. Gen Z, having grown up in a digital era, might display a higher level of comfort with AI interactions and an openness to seeking mental health support through such platforms.

Understanding the generational attitudes and perceptions toward AI-powered mental health virtual assistants is essential for tailoring these interventions to meet the diverse needs of different age groups. By uncovering the factors that contribute to or hinder acceptance, mental health practitioners, technologists, and policymakers can collaborate to develop virtual assistants that resonate with each generation. Moreover, insights from this study could inform the implementation of appropriate strategies to mitigate potential barriers and promote the use of AI-powered mental health virtual assistants as a valuable addition to the continuum of mental healthcare.

Literature review

The significance of mental health is multifaceted. It affects our daily lives. Mentally healthy people can manage stress, build good relationships, and achieve effectively in their personal and professional lives. It also affects cognition, decision-making, and environmental adaptation. Additionally, mental, and physical health are linked [8,9]. Mental health concerns such as depression and anxiety are linked to gastrointestinal, immunological, and cardiovascular ailments. Mental health activities such as sleep, exercise, and nutrition affect physical health [10-14]. Mental health is essential to society's well-being and cohesion. Social well-being, comprising social contacts, social ties, and community and belonging, is improved by mental health [15]. Untreated mental health difficulties can cause social marginalization, hurt relationships, higher healthcare expenses, and lower productivity [16,17].

Mental health is a fundamental human right. The World Health Organization regards mental health as a vital part of health and welfare [18] and believes everyone deserves the best mental health. Prioritizing mental health and providing proper services and support are crucial. The United Nations has included mental health in its sustainable development goals [19]. Mental disease has global repercussions. Anxiety, depression, substance use, bipolar, schizophrenia, eating disorders, obsessive-compulsive disorder, and post-traumatic stress disorder (PTSD) are common mental health illnesses [20]. Mental disorders afflict roughly one billion people, or 13% of the global population, according to 2019 statistics. At 31%, anxiety is the most common mental disorder, followed by depression at 28.9%. Development problems, attention deficit hyperactivity disorder (ADHD), and bipolar disorder are 11.1%, 8.8%, and 4.1% prevalent, respectively [21]. A study found that people with chronic mental illnesses die 10-20 years earlier than the normal population [22]. The data show that mental illness policy implementation is ineffective across socioeconomic groups. Only 3% of low-income, 13% of lower middle-income, 32% of upper-middle-income, and 25% of high-income nations reported 100% policy or plan compliance. This shows that low-income countries have a bigger problem [23]. Despite the severity of the issue, countries globally allocate only 2% of their health budgets to mental health [24].

Additionally, just 4.6% of global health research is dedicated to mental health [23], indicating a substantial dearth of interest and exploration in this area. Given the gravity of the issue, cost-efficient and effective mental health prevention and management treatments must be investigated. By viewing health literacy and support-based technology interventions as social practices rather than individual behavior change tactics, diverse community-based projects that improve health and equity can be developed. Health awareness and support programs at the social and policy levels are crucial. However, these policies must be carefully considered in light of their daily effects on individuals, businesses, and communities [25]. Community involvement is crucial to improving mental health services, especially when using advanced AI technologies in mobile apps.

AI technology has transformed the business and healthcare sectors. Automated virtual assistants or humanoids provide exact responses to user queries. AI technology solutions can improve diabetes self-management because of their simplicity, cost, information and instructions, engagement, and constant assistance. Advanced deep learning and natural language processing have led to broader language models such as AI-based virtual assistants. These models have been widely used in text production, language translation, and question-answering [26]. According to multiple research [27-29], AI-based virtual assistants outperform earlier models in accuracy and efficiency when answering a variety of questions. AI-based virtual assistants can also provide logically related and well-structured text for content generation and summarization [30].

AI-based virtual assistants can provide high-quality healthcare [31,32]. Teleconsultants can improve patient care and decision-making with AI-based virtual assistants. Teleconsultants can make better decisions with AI-based virtual assistants' timely and accurate information [33]. AI-based virtual assistants can also provide health information [34]. Previous research [35-39] has shown the importance of AI-based virtual assistants in healthcare. These studies also stressed the need for greater research to fully understand how AI-based virtual assistants affect patients' well-being. AI-based virtual assistants for mental health management have been shown to be effective [40-42]. A recent scoping analysis found that mental health virtual assistants have user-friendly interfaces, visually appealing designs, rapid response times, perceived reliability, and high user satisfaction [43]. There are few academic studies on AI assistants in medicine, especially on the various factors influencing their adoption [44]. Therefore, this study aims to analyze various influencing factors among generations X, Y, and Z of AI-powered mental health virtual assistants.

## Materials and methods

Study settings and participants

The data collection in this study employed a cross-sectional design to examine the many aspects that influence AI-powered mental health virtual assistants. The study sample consisted of outpatients diagnosed with various mental health illnesses, such as anxiety, depression, schizophrenia, and behavioral disorders. The participants were recruited from nine public hospitals located in Saudi Arabia. During the outpatient sessions, patients were invited to partake in the research study, during which the goal and objectives were elucidated. The commencement of the study was contingent upon obtaining prior authorization from the selected hospitals. The selected approach for facilitating patients' expression of thoughts and enhancing their comfort level involves the utilization of an online pre-validated survey questionnaire.

Recruitment and sampling

As the participants were purposively recruited from the selected hospitals, convenience and purposive sampling techniques were adopted [44]. The inclusion criteria included non-disabled patients who are aged above 18 years and have been using AI-powered mental health virtual assistants for not less than three months.

Instruments

The survey questionnaire is divided into two sections. The first section focuses on collecting demographic information related to age, gender, nationality, and experience with AI chatbots. The second section focuses on collecting the data on AI technology influencing factors. This study has adopted four factors including PE (four items), EE (three items), SI (three items), and FC (four items) from Alhwaiti and Kelly et al. [45,46]. In addition, BI (three items) was adopted from Joshi [47]. The questionnaire was designed using Google Forms, by creating a link to access the survey. A pilot study was conducted with 24 outpatients, and the data were analyzed. Cronbach alpha was calculated for all items and was observed to be greater than 0.7, indicating good internal consistency [48].

Ethical considerations

All the participants were fully informed about the study through an information sheet attached to the invitation email. An informed consent was taken from all the participants using a check button, before starting the survey. The participation was voluntary, and the participants were assured of their anonymity and their rights with respect to the data. Ethical approval (IRB-2023-03-328, Dt: 07/09/2023) was received from the Ethics Committee at Imam Abdulrahman Bin Faisal University, Dammam, Saudi Arabia.

Data collection

A participant information sheet is attached along with the invitation email (containing a survey link), explaining the rights of the participants, and forwarded to all the patients who agreed to participate in the survey. A total of 570 patients participated in the survey. However, 55 participants had no experience, and nine responses were incomplete. After cleaning the data, a total of 506 patients' responses were considered for data analysis.

Data analysis

To attain the objectives of the research, the researcher utilized Statistical Product and Service Solutions (SPSS, version 24) (IBM SPSS Statistics for Windows, Armonk, NY) for analyzing the data. Descriptive statistics were used to characterize the participants’ demographic data. In addition, two-sample t-tests with unequal variances and single-factor ANOVA were used to analyze the statistically significant differences among the participant groups from the data.

## Results

Participants' demographic information is presented in Table 1. About 56.3% of them are males. The majority of the participants (94.3%) were Saudi nationals. Participants in the age group of 18-26 years (Gen Z) included 38.5%, followed by 27-42 years (Gen Y: 32.4%), and greater than or equal to 43 years (Gen X: 29.1%).

**Table 1:** Participants' demographics

		Mean	Relative frequency
Age (in years)	18-26	195	38.5%
	27-42	164	32.4%
	>=43	147	29.1%
Gender	Male	285	56.3%
	Female	221	43.7%
Nationality	Saudi	477	94.3%
	Non-Saudi	29	5.7%

**Figure 1:**
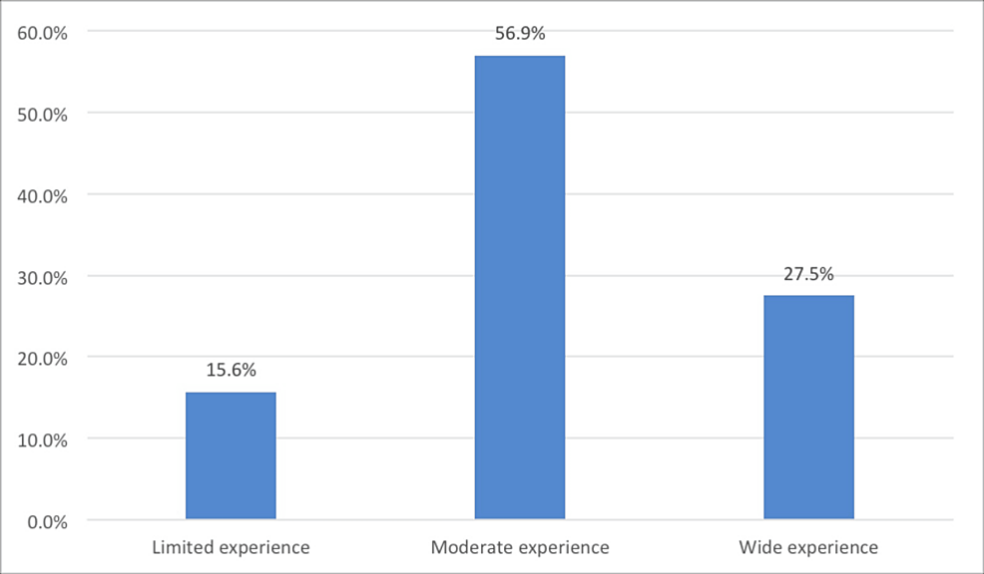
Participants’ experience of using AI-based mental health virtual assistants

As shown in Figure 1, the majority of the participants (56.9%) had moderate experience, followed by 27.5% having wide experience, and 15.6% having limited experience in using mental health AI-based virtual assistants.

Table 2 presents the measurements of influencing factors affecting the acceptance of AI-based mental health virtual assistants. The mean scores for performance expectancy factors are moderate, ranging from 2.79 to 3.06. It appears that users generally believe that AI chatbots have the potential to increase the efficiency of mental health processes, provide accurate information, improve the user experience, and enhance access to mental health-related information. This suggests that users perceive some value in AI chatbots for mental health support, but there is room for improvement. The mean scores for effort expectancy factors are also moderate, with scores ranging from 2.83 to 3.12. Users generally find interactions with AI chatbots to be relatively easy and convenient, indicating that AI chatbots are seen as user-friendly and not overly demanding in terms of effort. The score of 3.12 for the statement about AI chatbots becoming easier to use with more interactions suggests a belief in the potential for these systems to become even more user-friendly over time. Social influence factors have relatively lower mean scores, with scores ranging from 2.51 to 2.84. While some users feel motivated to use AI chatbots due to societal acceptance, the influence of friends and family appears to be weaker. This indicates that the opinions of peers and significant others may not be strong drivers of AI chatbot adoption in the context of mental health support. Facilitating conditions, including the availability of technical resources, knowledge, peer support, and previous experience, also received moderate mean scores, ranging from 2.68 to 2.81. Users generally have some level of access to the necessary resources and support to use AI chatbots, although there is room for improvement in terms of enhancing facilitating conditions. The mean scores for behavioral intention factors are moderate, ranging from 2.87 to 2.98. Users express an intention to use AI chatbot services for mental health assistance in the future, provided that certain conditions are met, such as long-term availability and reliability.

**Table 2:** Influencing factors of AI-based mental health virtual assistants acceptance

Measurements of the Factors	Statements	Mean
Performance Expectancy (PE)	AI chatbots increase the efficiency of the processes related to mental health services	2.99
	AI chatbots provide accurate information about mental health management, care, and services	2.79
	AI chatbots improve the overall user experience related to mental health management, care, and services.	2.92
	AI-enabled chatbots improve access to information about mental health management, care, and services	3.06
Effort Expectancy (EE)	My interaction with AI chatbots provided easy-to-understand information	2.94
	Interacting with AI chatbots takes a minimal amount of physical and mental effort when it comes to mental health management, care, and service related tasks	2.83
	AI chatbots are easier to use and I think can learn to be proficient with more interactions	3.12
Social Influence (SI)	People who are important, like friends and family, believe that I should take help from AI chatbots	2.63
	People who can influence my behaviors like peers think I should use AI chatbots	2.51
	Acceptance of AI chatbots and technologies within society has motivated me to use them	2.84
Facilitating Conditions (FC)	I have the resources such as technical assistance and devices to use AI chatbots	2.72
	I have the basic knowledge and expertise to use AI chatbots effectively	2.7
	I have peer support who can assist me in using AI chatbots	2.81
	I have previous experience using similar AI chatbot services for other purposes	2.68
Behavioral Intention (BIs)	I intend to use AI chatbot services to get assistance and help in the future	2.98
	Given the opportunity, I would use AI-powered chatbots for obtaining reliable information	2.87
	If AI chatbots availability is assured for long-term, I would be using it for information needs	2.92

Table 3 provides an insightful analysis of participants' perceptions categorized by gender across key variables related to the acceptance of AI-based mental health virtual assistants. In terms of performance expectancy, the mean scores for both males and females are relatively close, with males at 2.97 and females at 2.91, suggesting that both genders perceive AI-based mental health virtual assistants as having a similar potential to enhance performance in mental health-related tasks. Similarly, examining EE, SI, FC, and BI, the pattern remains consistent. The analysis of Table 3 indicates that gender does not play a significant role in shaping perceptions of AI-based mental health virtual assistants across the variables of PE, EE, SI, FC, and BI. Both male and female participants generally share similar views and expectations regarding the use of AI technology for mental health support.

**Table 3:** Participants' perceptions differ by genders SD: standard deviation; df: degrees of freedom; N: number of participants

	Variables	N	Mean	SD	df	t-value	p-value
Performance expectancy	Male	285	2.97	0.84	486	0.73268	0.2321
	Female	221	2.91	0.73			
Effort expectancy	Male	285	2.99	0.78	469	0.82684	0.2043
	Female	221	2.92	0.81			
Social influence	Male	285	2.71	0.97	489	1.8583	0.1181
	Female	221	2.6	0.83			
Facilitating conditions	Male	285	2.77	0.89	480	1.2486	0.1061
	Female	221	2.67	0.83			
Behavioral intentions	Male	285	2.98	0.85	484	1.3749	0.0848
	Female	221	2.86	0.76			

Table 4 provides a comprehensive insight into how different age groups perceive AI-based mental health virtual assistants across various influencing factors. In terms of PE, Gen Y stands out with the highest average score, indicating the most positive perception of how AI-based mental health virtual assistants can enhance performance, closely followed by Gen Z. Gen X, on the other hand, shows a notably lower average, suggesting a less optimistic view of these systems. In terms of EE, both Gen Z and Gen Y have relatively high averages, suggesting that these generations find AI-based mental health virtual assistants easy to use and not overly demanding. Gen X, however, exhibits a lower average, indicating a relatively higher perception of the effort required. In terms of SI, Gen Y perceives a somewhat stronger influence from their social circles regarding the use of AI-based mental health virtual assistants, while Gen Z follows closely. Gen X has the lowest average in this regard, implying a lesser impact of social influence on their intentions. In terms of FC, Gen Z and Gen Y have similar perceptions of access to resources and support for using AI-based mental health virtual assistants, while Gen X scores lower, suggesting a comparatively less favorable perception of available facilitating conditions. In terms of BI, Gen Y displays the highest average, indicating a strong intention to use AI-based mental health virtual assistants in the future. Gen Z closely follows, while Gen X exhibits the lowest average in this aspect, indicating a lesser inclination toward future use.

**Table 4:** Summaries of influencing factors by age groups (Gen X, Y, and Z)

	Groups	Count	Sum	Average
Performance expectancy	Gen Z	195	599.25	3.073077
	Gen Y	164	512.75	3.126524
	Gen X	147	378.5	2.57483
Effort expectancy	Gen Z	195	605	3.102564
	Gen Y	164	500.3333	3.050813
	Gen X	147	395.6667	2.69161
Social influence	Gen Z	195	528	2.707692
	Gen Y	164	461	2.810976
	Gen X	147	357.6667	2.433107
Facilitating conditions	Gen Z	195	565.25	2.898718
	Gen Y	164	472.25	2.879573
	Gen X	147	345.25	2.348639
Behavioral intentions	Gen Z	195	605.6667	3.105983
	Gen Y	164	514.3333	3.136179
	Gen X	147	362.3333	2.464853

Table 5 presents the results of the analysis of variance (ANOVA) test, which assesses the differences in influencing factors among different age groups. The ANOVA results for PE, EE, SI, FC, and BI indicate that there are statistically significant differences (p < 0.05) between the age groups.

**Table 5:** ANOVA results for influencing factors by age groups (Gen X, Y, and Z) df: degrees of freedom; *: statistically significant; SS: sum of squares; MS: mean of squares

	Source of Variation	SS	df	MS	F	P-value	F crit
Performance expectancy	Between Groups	28.74529	2	14.37265	19.35541	< 0.0001*	3.013645
	Within Groups	373.5101	503	0.742565	-		
Effort expectancy	Between Groups	15.88393	2	7.941963	10.37641	< 0.0001*	3.013645
	Within Groups	384.9894	503	0.765386	-		
Social influence	Between Groups	11.74836	2	5.87418	6.588578	0.001498*	3.013645
	Within Groups	448.4598	503	0.89157	-		
Facilitating conditions	Between Groups	30.59521	2	15.29761	18.83342	< 0.0001*	3.013645
	Within Groups	408.566	503	0.812258	-		
Behavioral intentions	Between Groups	44.81585	2	22.40792	30.69281	< 0.0001*	3.013645
	Within Groups	367.2257	503	0.730071	-		

## Discussion

The purpose of this study is to identify the influencing factors associated with AI mental health virtual assistant acceptance. Accordingly, more than one-third of the participants in this study had moderate to wide experience of using these assistants, indicating the wider use of such assistants in their daily lives. However, a recent study [49] on COVID-19-related chatbots in Saudi Arabia has observed that participants' awareness of using health-related chatbots was low, but most of them held positive perceptions of these AI-based chatbot assistants and were willing to use them in different areas such as health awareness and mental-health-related issues such as anxiety and depression due to COVID-19. An increase in the use of AI-enabled virtual assistants, as observed in this study, can be related to the increase in the use of virtual assistants during the pandemic. In relation to performance expectancy, access to mental health information and increased efficiency of mental-health-related services were identified to be the major factors, which were supported by the findings in two studies [50,51]. However, both these studies highlighted the issues of ethical and privacy concerns involved in mental health support, which may influence the acceptance of AI-based virtual assistants. From the findings, it is observed that AI virtual assistants are easier to use and provide easy-to-understand information. Recent studies [52,53] observed that Arabic language chatbots driven by the latest technologies using AI and NLP are relatively scarce largely due to the complex nature of the Arabic language, and the AI virtual assistants are still lagging in applying the Arabic language in AI-based assistant development in the fields of mental health. However, with the adoption of innovative techniques such as deep learning, recent studies [54,55] have observed greater accuracy of the Arabic language in virtual assistants, indicating the increased ease of use and understandability of information provided by the assistants.

Findings on social influence suggested that greater acceptance among society has motivated the participants to use AI virtual assistants similar to the findings of Henkel et al. [56]. It was observed that previous experience with assistants had a moderating effect on the relationship between social influence and intention to use [56]. This indicates that, while social influence can be a significant influencing factor, individuals' skills and experience can also influence the adoption of AI virtual assistants. Accordingly, the findings related to the facilitating conditions reflected the availability of skills, expertise, and resources as the important factors enabling them to use virtual assistants. Focusing on the intention to use, using for information needs and reliable information were the strong reasons for the continued use of AI virtual assistants. Recent studies [50,57-59] exploring other factors have identified factors such as anthropomorphism, trust, and assistants' personality traits to be influencing the acceptance of AI virtual assistants, indicating the emergence of various factors in continuing research. For instance, a trusting bond was developed within four days between a company chatbot and its users, which may take days with human therapists [57]. Although AI-enabled solutions demonstrate potential in the field of mental health, further research is required to examine the ethical and societal implications of these technologies. Furthermore, it is necessary to build effective research and medical practices within this innovative area [50]. Overall, findings suggested that acceptance does not only depend on the design of the chatbot software but also on the characteristics of the user, and most prominently on self-efficacy, state anxiety, learning styles, and neuroticism personality traits.

The findings highlighted the variations in how different generations perceive AI-based mental health virtual assistants, with Gen Y generally having more positive perceptions and stronger intentions, Gen Z closely aligned with them, and Gen X demonstrating relatively lower expectations and intentions. The ANOVA results emphasize the influence of age on perceptions of various influencing factors related to AI-based mental health virtual assistants. Although existing research on influencing factors of AI virtual assistants for mental health is limited, findings from similar studies in other areas can provide valuable insights. For instance, a recent study [60] has found that 70% of Gen Z users are using AI technology, but only 52% of them trust it. It also found that among the non-users, 68% are Gen X, 88% are unclear about the impact of AI on their life, and 40% are not familiar with AI technology. These findings are similar to the findings in this study, which has identified significant differences among the Gen X and Gen Z users of mental health AI virtual assistants. Similar results were observed in relation to the greater preference among Gen Z toward customer service virtual assistants [61] and AI-education assistants [62] compared to Gen X users. However, contrasting results were observed in the area of AI-enabled Internet banking assistants, where Gen Z did not perceive any advantage in using them [63]. In a different context, a recent study [64] has observed that Gen Z users had little knowledge or experience with AI systems, but they typically had a favorable opinion of AI-based virtual assistants for health insurance. Similarly, a cross-cultural study [65] including 48 countries and eight regions observed the significant influence of socio-cultural factors, such as religions and regions on the acceptance of non-conscious data collection of AI assistants, indicating the varying perceptions of factors such as privacy and security associated with AI assistants. For example, in a study [66] conducted in the USA, 48% of Americans said that they would share their medical history with AI to help support their care needs, compared to 65% of millennials. These contrasting findings from the various studies suggest that human-machine interaction is part of a complex process in which there are different elements determining individuals’ acceptance of the use of AI devices during daily life, especially in relation to mental health [67]. However, creating awareness and inducing motivation can foster the use of AI virtual assistants [68].

The findings of this study have significant theoretical and practical implications in the context of AI-powered mental health virtual assistants. The theoretical implications underscore the importance of recognizing generational differences in technology acceptance. Understanding how different age groups perceive these AI solutions helps in refining existing technology acceptance models and theories. Notably, the study reaffirms the influence of PE, EE, SI, FC, and BI in shaping the acceptance of AI chatbots. Practically, these findings suggest that developers, mental health practitioners, and policymakers should take a nuanced approach to tailor AI-powered mental health virtual assistants to meet the distinct needs of Gen X, Y, and Z. This can involve addressing privacy concerns, offering user education, leveraging social marketing, ensuring users have necessary support and resources, and committing to long-term availability. Additionally, ethical considerations should remain a priority, as trust and data security are pivotal in the successful adoption of these virtual assistants. Overall, the study sheds light on the multifaceted factors influencing the acceptance of AI in mental healthcare and provides actionable insights for improving these technologies to meet the diverse needs of different generations.

Despite its valuable insights, this study also presents certain limitations that should be considered when interpreting its findings. First, the study's sample was drawn from outpatient populations in Saudi Arabia, which might not be representative of the broader global population. Consequently, the cultural and regional specificities of the sample may limit the generalizability of the results to other cultural contexts. Second, the study relied on self-reported data from participants, which can introduce response bias and social desirability bias, potentially influencing the accuracy of the findings. Furthermore, the study's cross-sectional design provides a snapshot of participants' attitudes and perceptions at a single point in time, making it challenging to capture potential changes in these attitudes over time. Longitudinal studies could provide a more comprehensive understanding of the evolution of acceptance over time. Additionally, the study focused on the factors influencing acceptance but did not delve deeply into the potential impact of the virtual assistant's effectiveness on actual mental health outcomes, which is a critical aspect of their utility. Finally, the study did not explore the potential disparities in access to technology or digital literacy among different age groups, which can significantly affect their ability to use AI-powered mental health virtual assistants effectively. Future research can address these limitations and offer a more comprehensive understanding of the complexities surrounding the acceptance and use of AI in mental healthcare.

## Conclusions

This empirical study on the acceptance of AI-powered mental health virtual assistants among different generational groups, including Gen X, Y, and Z, sheds light on the multifaceted factors influencing the adoption of these innovative technologies. The findings underscore the significance of considering generational differences in attitudes and perceptions, with Gen Y and Gen Z demonstrating more positive attitudes and stronger intentions to use AI mental health virtual assistants, while Gen X appears to be more cautious. The study highlights the importance of factors such as PE, EE, SI, FC, and BI in shaping acceptance. These findings contribute to our understanding of technology acceptance models and highlight the need for tailored approaches to cater to diverse generational preferences. Further research and practical applications can build upon these insights to enhance the role of AI in mental healthcare.
